# Circumventing ‘free care’ and ‘shouting louder’: using a health systems approach to study eye health system sustainability in government and mission facilities of north-west Tanzania

**DOI:** 10.1186/s12961-016-0137-9

**Published:** 2016-09-09

**Authors:** Jennifer J. Palmer, Alice Gilbert, Michelle Choy, Karl Blanchet

**Affiliations:** 1 Department of Infectious Diseases Epidemiology, Faculty of Epidemiology and Population Health, London School of Hygiene & Tropical Medicine, Keppel St, London, WC1E 7HT United Kingdom; 2 Centre of African Studies, School of Political and Social Sciences, University of Edinburgh, Edinburgh, United Kingdom; 3 Department of Clinical Research, Faculty of Infectious and Tropical Diseases, London School of Hygiene & Tropical Medicine, Keppel St, London, WC1E 7HT United Kingdom; 4 Faculty of Public Health and Policy, London School of Hygiene & Tropical Medicine, 15-17 Tavistock Pl, London, WC1H 9SH United Kingdom; 5 Department of Global Health and Development, Faculty of Public Health and Policy, London School of Hygiene & Tropical Medicine, 15-17 Tavistock Pl, London, WC1H 9SH United Kingdom

**Keywords:** Eye care, Eye health system, Sustainability, Faith-based organisations, Tanzania, Cataract surgeon, User fees, Health systems research

## Abstract

**Background:**

Little is known about the contributions of faith-based organisations (FBOs) to health systems in Africa. In the specialist area of eye health, international and domestic Christian FBOs have been important contributors as service providers and donors, but they are also commonly critiqued as having developed eye health systems parallel to government structures which are unsustainable.

**Methods:**

In this study, we use a health systems approach (quarterly interviews, a participatory sustainability analysis exercise and a social network analysis) to describe the strategies used by eye care practitioners in four hospitals of north-west Tanzania to navigate the government, church mission and donor rules that govern eye services delivery there.

**Results:**

Practitioners in this region felt eye care was systemically neglected by government and therefore was ‘all under the NGOs’, but support from international donors was also precarious. Practitioners therefore adopted four main strategies to improve the sustainability of their services: (1) maintain ‘sustainability funds’ to retain financial autonomy over income; (2) avoid granting government user fee exemptions to elderly patients who are the majority of service users; (3) expand or contract outreach services as financial circumstances change; and (4) access peer support for problem-solving and advocacy. Mission-based eye teams had greater freedom to increase their income from user fees by not implementing government policies for ‘free care’. Teams in all hospitals, however, found similar strategies to manage their programmes even when their management structures were unique, suggesting the importance of informal rules shared through a peer network in governing eye care in this pluralistic health system.

**Conclusions:**

Health systems research can generate new evidence on the social dynamics that cross public and private sectors within a local health system. In this area of Tanzania, Christian FBOs’ investments are important, not only in terms of the population health outcomes achieved by teams they support, but also in the diversity of organisational models they contribute to in the wider eye health system, which facilitates innovation.

**Electronic supplementary material:**

The online version of this article (doi:10.1186/s12961-016-0137-9) contains supplementary material, which is available to authorized users.

## Background

Christian missionaries and faith-based organisations (FBOs) have long played a prominent role in health systems in Africa. During the colonial period, Christian missions were the cornerstone for the promotion of Western-style health, education and wealth. Today, they derive much of their legitimacy as organisations capable of reaching the ‘grass roots’ with their continued relevance attributed to the failures of the African State to deliver services and engage holistically with poor peoples’ needs [1]. This contribution is widely acknowledged in high-level global health policy dialogue and practice, for example, through the awarding of large development grants to Christian FBOs by major donors [2]. Such FBOs may deliver services themselves and/or coordinate aid to the health programmes of local church congregations which run health facilities. However, surprisingly little is known about the comparative benefits, harms and contributions of faith-based, non-state actors alongside government providers in achieving the basics of universal health coverage (health system reach to poor people, cost to patients and satisfaction of patients with services) or the sustainable health system governance that supports this [2–5].

Reproductive and sexual health problems (e.g. HIV/AIDS, contraception) have provided rich terrain for other scholars investigating the influence of Christian FBOs and churches on African development because of assumed tensions between religion and the linked concepts of development/modernity/liberalism [1]. Initially, church organisations tended to deny HIV or condemn HIV responses; but as HIV grew into a large-scale social and moral crisis, participation in mainstream international health responses became a way for many Church organisations in Africa to confront the social stigma and structural inequalities that shape the epidemic and was an overtly religious and political act [6]. Today, the political economy of HIV engagement can be as beneficial to Christian FBOs and churches (such as for organisational survival) as to a Church’s parishioners and patients [7]. Blindness, on the other hand, has commonly been an attractive and noble health pursuit for religious charities in Africa, but little is known about what eye health does ‘for’ Christian FBOs, churches and the health systems they are a part of. In this paper, we argue that a very narrow and specialist health concern – eye care – provides an interesting alternative case study of the role of faith-based providers in African development because of the relative invisibility of this problem to the State.

As with other health problems, early eye care work in many parts of Africa (Ghana (1930s), Nigeria (1940s–50s), Tanzania, Uganda and Malawi (1960s), for example), was performed by missionary physicians [8]. Although secular international and African non-governmental organisations (NGOs) probably dominate eye care development work on the continent today, Christian faith continues to motivate several large eye health development actors as symbolised by the Vatican’s role as host of a 2012 meeting to discuss the future of international eye health [9, 10].

One in seven eye care practitioners in sub-Sahara Africa (19% of eye surgeons) work in a facility run by NGOs (including Christian FBOs) [11]. NGOs and FBOs also often provide the bulk of funding, equipment and consumables in national eye care programmes [12–16], with positive effects on the productivity of eye health workers [16]. Eye care NGOs and FBOs work in ways that may be judged more or less sustainable, ranging from decades-long programmatic support for specific hospitals to short-term expatriate-led missions to distribute recycled spectacles [17–19].

A consistent critique has emerged over the last decade, however, which charges NGOs and FBOs with having developed eye health systems parallel to government structures that must be maintained through continuous external financing, leaving eye health as a neglected area of development by African governments [10, 16, 20–22]. Opinion leaders have therefore called for a “*paradigm shift*” [20] (p. 4) in the way eye care NGOs/FBOs work, calling for closer and better coordination at sub-regional and country levels. In particular, the International Agency for the Prevention of Blindness and the WHO-led VISION 2020 campaign encourage national eye health coordinators within Ministries of Health and NGOs/FBOs to advocate for greater, long-term domestic support to eye care and better integration of NGO/FBO eye care services into national health systems [23–25]. From the perspective of NGOs/FBOs, following the VISION 2020 approach appears to require a shift in programming emphasis. If “*service delivery alone will not bring about systematic change*” [26] (p. 70), then NGOs/FBOs must begin to engage with “*society and systems*” [10] (p. 74). Additionally, while many NGOs/FBOs seek to keep a hand in direct service delivery (either to maintain financial contributions from private donor bases or to maintain credibility in advocacy work [10, 26]), for others, this policy position inevitably means a reduction in support to this type of on-the-ground work or to specific components of it such as outreach activities.

With some exceptions [15, 27], scant literature has explored how eye health personnel define and work towards sustainability in this context, across the parallel NGO/FBO and government systems that VISION 2020 seeks to bring together. A health policy and systems approach has been suggested by scholars to interrogate the complicated relationships between religion, public health and development [2, 28]. In this study, we use a health systems approach to describe the strategies used by eye care practitioners in four hospitals of north-west Tanzania to navigate the government, mission, FBO and donor rules that govern eye services delivery here. We furthermore seek to understand the entry and potential contribution of a new pan-sectoral and informal eye care practitioners’ network, the Lake Region Eye Care Services Association (LARESA).

Before a note on methodology, we review literature on the policy context of government, Christian faith-based and secular NGO engagement in eye health in Tanzania, and define our study setting more specifically. We then examine how practitioners characterise service delivery in each sector and analyse service output in relation to observed models of financing, service diversification and use of social capital. We end by suggesting how actors in Tanzania can better conceptualise and contribute to eye health system sustainability to achieve equitable development across the sectors.

### Eye health development in Tanzania

Christian missionaries from Germany, Britain, Sweden and other European countries were responsible for much of the earliest modern health infrastructure in inland, rural Tanzania [29]. The first cataract surgeries were probably carried out at Mvumi mission hospital in the 1930s by general surgeons (Allen Foster, personal communication) and this was also the first hospital in the country to employ an ophthalmologist in the 1960s [8]. By the time of independence in 1961, Christian missionary societies owned 42% of all hospital beds in the country and 81% of the primary healthcare facilities [30].

Following an ‘African socialist’ approach to development, President Nyerere nationalised many mission health facilities beginning in the late 1960s [30, 31]. As a leading advocate of the WHO 1978 Alma Ata declaration, Tanzania later took steps to divert resources from hospitals into front-line facilities [31]. This broad approach may have indirectly supported community eye care services, through local government recognition of the eye health needs of elderly people for spectacles in high-profile adult literacy campaigns [32, 33]. Financial contributions to fund spectacle donations, for example, were encouraged from local groups in the spirit of self-reliance [32].

During this period, specialist services, such as eye care, created important symbolic justification for retaining mission actors in the health system. In the early 1970s, Nyerere commissioned Church missions, supported by international FBOs, to build regional teaching and referral hospitals to house specialist services [29]. These institutions were responsible for training the country’s first non-physician assistant medical officers in ophthalmology (AMOOs), ophthalmologists and optometrists between 1975 and 1985 (Nkundwe Mwakyusa and Allen Foster, 2012, personal communications). With the global economic crisis and the imposition of World Bank structural adjustment programmes in the 1980s, many services at government-run district and referral hospitals became run-down [29, 31]. This created political space for domestic Church missions to retake ownership of key hospitals in the country [30, 31, 34] and reinvigorated FBO, NGO and donor interest in specialist hospital services, including eye care [35].

Healthcare in Tanzania was further reformed in the 1990s through a process of decentralisation and the adoption of patient user fees and insurance mechanisms [36–38]. Development funding was reorganised into a more flexible sector-wide approach, allowing the Ministry of Health and Social Welfare access to greater resources to develop the hospital system and re-engage government–voluntary sector partnership models. High-performing hospitals, regardless of the sector they are in, may now apply for additional government funding as district designated hospitals. If it is an NGO or Church-run facility, it becomes co-managed by district government structures and has access to central government funds for staff salaries and district ‘basket’ funds for medical supplies and infrastructure [36]. This normally represents a financial opportunity for many Church-owned facilities which, in recent years, have seen a decrease in direct donor funding due to the sector-wide approach or other causes [30]. At least nine international eye care NGOs or Christian FBOs operate in Tanzania today [39].

## Methods

### Setting and selection of study sites

The ‘Lake Region’^1^ of north-west Tanzania, where this study was conducted, has far fewer human resources for eye health and performs less cataract surgeries than recommended for sub-Saharan Africa (0.2 ophthalmologists per million population compared to a target of 4.0; 6.1 mid-level personnel including AMOOs and nurses compared to the 10.0 target; 1.4 optometrists compared to a 20.0 target; data collected from districts by study team, see also [11]). At the time of this study, the Lake Region was populated by more than 10 million people but had only one surgically-active ophthalmologist. Non-physician AMOOs and cataract surgeons (AMOOs with an extra year of training in cataract and other minor eye surgeries) therefore lead development of the sector here, delivering the most complex eye health interventions in the area. Six out of 11 cataract surgeons/AMOOs were based in mission-owned facilities. There were 11 hospitals owned by church missions in the Lake Region (including Lutheran, Catholic and Africa Inland Church denominations), compared to 29 fully government-run facilities and around 15 private for-profit hospitals or practices.

We purposively selected four cataract surgeons and their teams from four different regions to follow prospectively based on geographic accessibility for the study team and sector of employment: two from the government sector (Government Hospitals A and B) and two from the mission sector (Mission Hospitals A and B). Government Hospital B received additional support from a secular international eye health NGO. Mission Hospital A was a district designated hospital and was co-owned by the government. Those from the mission sector were supported by the funder of this study, an international faith-based organisation, Christoffel-Blindenmission (CBM), which has been supporting eye health services in Tanzania since 1973. We also followed regional developments in eye care through study of an eye care practitioner’s organisation which all surgeons in the study were members of, LARESA.

The private for-profit sector is relatively small in the Lake Region but slowly growing in terms of ophthalmologic services (such as cataract surgery) in Tanzania, and is the main provider of optometric services, but we did not have space to explore private practice dynamics here.

### Field work

Field work consisted of four quarterly field visits to the Lake Region lasting 2–5 weeks each over a 1 year period (September 2012 to August 2013). Data was collected by a team of four expatriate researchers who contributed information to a central database, with one (JP) collecting data from all case study hospitals, two (AG and MC) spending extended periods in two case study hospitals each, and another (KB) focusing on study of LARESA and the sustainability analysis workshop. Data collection was prioritised in the four case study hospitals but also occurred opportunistically in six others to further contextualise our analysis across all LARESA areas.

On each visit, qualitative data was collected through in-depth interviews with staff from eye departments, hospital management teams, mission health programmes, and district and regional medical offices. Interviews were audio-recorded and followed a loose topic guide that addressed the following issues: the history and future plans of the eye programme at each hospital; emergent events, relationships between actors of the health system, management decisions, and associated rationale affecting eye care in the last quarter; and local perceptions of eye health system sustainability. Interview data was supplemented with field notes from informal discussions during observations of eye care activities and review of documents relating to eye care activities in the hospital.

Each quarterly field visit additionally pursued a specific complementary data collection objective: (1) a mapping exercise to identify all eye care human resources and programmes in the Lake Region; (2) a participatory sustainability analysis exercise conducted with LARESA members, individuals involved in government and NGO eye health system planning, and patient representatives, to choose indicators of eye health system sustainability in the Region and obtain measures in the case study hospitals (process adapted from [40, 41]; see [42] for details on methods, indicators selected and baseline measurements); (3) a social network analysis to identify the range and types of actors who support eye care at each case study hospital and in which domain (e.g. reporting, outreach, equipment procurement, human resources recruitment, advocacy, etc., using methods described in [43]); and (4) close observation of decision-making surrounding the planning of outreach activities. Study of LARESA also involved interviews with members and the chairperson, observation of one meeting, and review of meeting minutes and other documents since its establishment.

All notes, transcripts, documents and other research products were imported into a central NVivo qualitative analysis database and coded line-by-line to understand broad eye health sustainability narratives in the Lake Region. All information collected on individual case studies was assembled to understand the evolution of three main types of activities which emerged in discussions and tended to dominate enquiries on sustainability in each hospital: departmental financing, how services became diversified, and how information was shared among peers. After this internal process was completed for each case study, experiences were compared across hospitals and any differences by sector (mission vs. government) were identified and further explored [44]. Finally, by analysing theoretical eye health sustainability narratives alongside our observations of the processes of change pursued by eye departments in practice, we were able to identify four thematic ‘sustainability strategies’, which explained the most important ways in which eye care actors work to achieve sustainability in the Lake Region eye health system.

## Results

### Eye care: a ‘blind spot’ for government

Eye health sustainability was most commonly perceived by actors in the Lake Region as constrained by systemic government neglect. At the sustainability workshop, each national- and regional-level actor who was asked to contribute information for a contextual overview of the eye care system emphasised low government prioritisation of funds for eye health as a reason why eye health human resources, infrastructure, equipment and activities were deficient in hospitals [42]. These deficiencies subsequently compromised implementation of Tanzania’s domestic eye health strategies. Partly, this was seen as a problem of global and therefore domestic health priorities: by not directly causing mortality, vision loss could not compete with interventions for maternal-child health and HIV prioritised by the Millennium Development Goals, which play a key role in framing the health strategy in Tanzania.

At regional level, while regional eye care coordinator positions existed, actors pointed to their typical exclusion from formal regional health management teams. Consequently, local advocacy efforts aimed at these structures by eye care practitioners themselves were considered ineffective (data obtained from a sustainability workshop presentation by LARESA chairperson).

Absence of basic eye care commodities in national supply systems was also strongly symbolic of neglect in this system for eye care actors; one cataract surgeon described being able to buy such items from the national medical store rather than from more expensive private sources, as a “*dream*” (interviews with eye care practitioners (ECPs) in Mission Hospital B and Government Hospital A). The ambivalent procurement strategy in the current national eye care plan, whereby closer links to the national medical store would be sought alongside development of new processes to purchase outside of it, also perhaps highlights the plan’s authors’ mistrust of wider government willingness to prioritise eye care [45]. A common saying among eye care practitioners therefore was, “*the government has a blind spot on eye services*” (ECP in non-case study hospital).

At the hospital level, general managers themselves admitted they often forgot about the equipment needs of this specialty service because they had little exposure to eye health in medical training. Furthermore, practitioners felt there was little public appetite to address eye health needs: “*If the maternal mortality rate goes up, the politicians will come here and ask why. But if many patients become blind nobody will care*” (ECP, Government Hospital A).

### Eye care: ‘All under the NGOs’

Given the relative lack of engagement by government in eye care in Tanzania, key national-level eye health system actors therefore characterised eye care as “*all under the NGOs*” (presentation by national eye care programme representative), and a sector in which donor assistance would always be required (interview with eye health donor). Throughout the study period, donors were an obvious resource to whom cataract surgeons were consistently referred when seeking funding for routine eye care activities from hospital, district, regional and even national management teams. Donor support was also seen as the only way eye services could ever be provided free to patients (ECPs, Government Hospital A and B). However, eye care actors also saw NGO funding as inherently precarious. For instance, in all annual reports written by CBM-funded eye departments to hospital management during the study period, surgeons highlighted the challenge of working when donor funds were “*late and insufficient*” (Mission Hospital A Annual Report 2012); workshop attendees therefore selected two indicators related to this topic to measure process towards system sustainability (Additional file 1) [42].

### A sectoral divide?

Analysis of key population outcomes in the case study hospitals painted a mixed picture of programme performance across the government and mission sectors.

In terms of the total number of eye patients examined by teams in 2012 (Table 1), no clear association could be identified with the hospital sector that eye care teams worked in.^2^ On the other hand, the number of eye surgeries that teams performed may have been associated with the sector, since both mission hospitals performed more surgeries than both government hospitals (969 and 985 vs. 0 and 605, respectively, considering those conducted both at the facility and by outreach). The government hospital with external eye care donor funding also notably performed more surgeries than the government hospital with none.

**Table 1:** Key population health outcomes by case study hospital in 2012

	Government A	Government B	Mission A	Mission B
Number of eye patients examined by team				
At base	2599	5020	7180	1720
Via outreach	0	4682	12,502	161
Total	2599	9702	19,682	1881
Number of cataract surgeries performed by team				
At base	0	605	498	955
Via outreach	0	0	471	30
Total	0	605	969	985
Number of cataract surgeries performed by other surgical teams in hospital’s region				
Total	334	0	254	0
Regional CSR				
All teams	146	347	441	642

*CSR* cataract surgical rate, per million population, using 2012 population data

The remaining sections of this paper will seek to explore the dynamic factors that contributed to the delivery of these key eye care services, paying particular attention to the strategies teams undertook to improve or maintain their sustainability. Four main strategies are discussed.

### Sustainability strategy 1: maintain ‘sustainability funds’

Apart from the government and donor revenue sources mentioned in dominant sustainability narratives, patient fees also offered an important stream of revenue to hospital eye departments and were accounted for in complex ways in eye care actors’ considerations of system sustainability.

In the short term, eye care actors saw collection of user fees as the most practical way to overcome financial deficiencies in government structures and dependency on NGOs to achieve sustainability. When patient fees were collected and/or managed by ECPs themselves, this type of income was “*easy to get*”, it was reliable given the greater demand for services than supply could provide and, through short-term forecasting, practitioners could tell from patient numbers when they would “*need to pull up* [their] *stockings*” to bring in more patients and therefore revenue through outreach (ECP at Mission Hospital B). Furthermore, this type of income could be used very flexibly. Access to this type of income normally translated into greater autonomy for eye teams as they did not need to seek permission from actors outside the eye team, such as hospital managers or donors, for regular purchases or for those that required quick decisions. Income from user fees could also be used to purchase drugs outside the national medical store – something which could not easily be done with money coming through typical government sources dedicated to purchasing in federal systems. During the indicator measurement exercise, when the team from one government hospital calculated the proportion of the eye department budget which came from patient fees at 36%, they were disappointed, seeing this as unsustainable; 70% was their ideal so that they would not have to depend on the government.

Income from patient fees was also commonly referred to using a meaningful colloquial name by eye care actors: the ‘sustainability fund’. It was a reserve fund that teams could build up slowly and protect, since, “*every fund generated in the hospital, has to go to the common pool, right? From the common pool, the management committee of the hospital decides oh we need this and we need this and then there is no money, there are no funds* [left] *for eyes*” (ECP at non-case study hospital). Additionally, with a small, departmentally-controlled fund, this could help eye teams demonstrate to others (e.g. hospital management, local government or donors) that they wanted to “*progress*” or build their unit’s sustainability, indirectly encouraging external actors to “*pitch in*” (*ibid*).

**Fig. 1:**
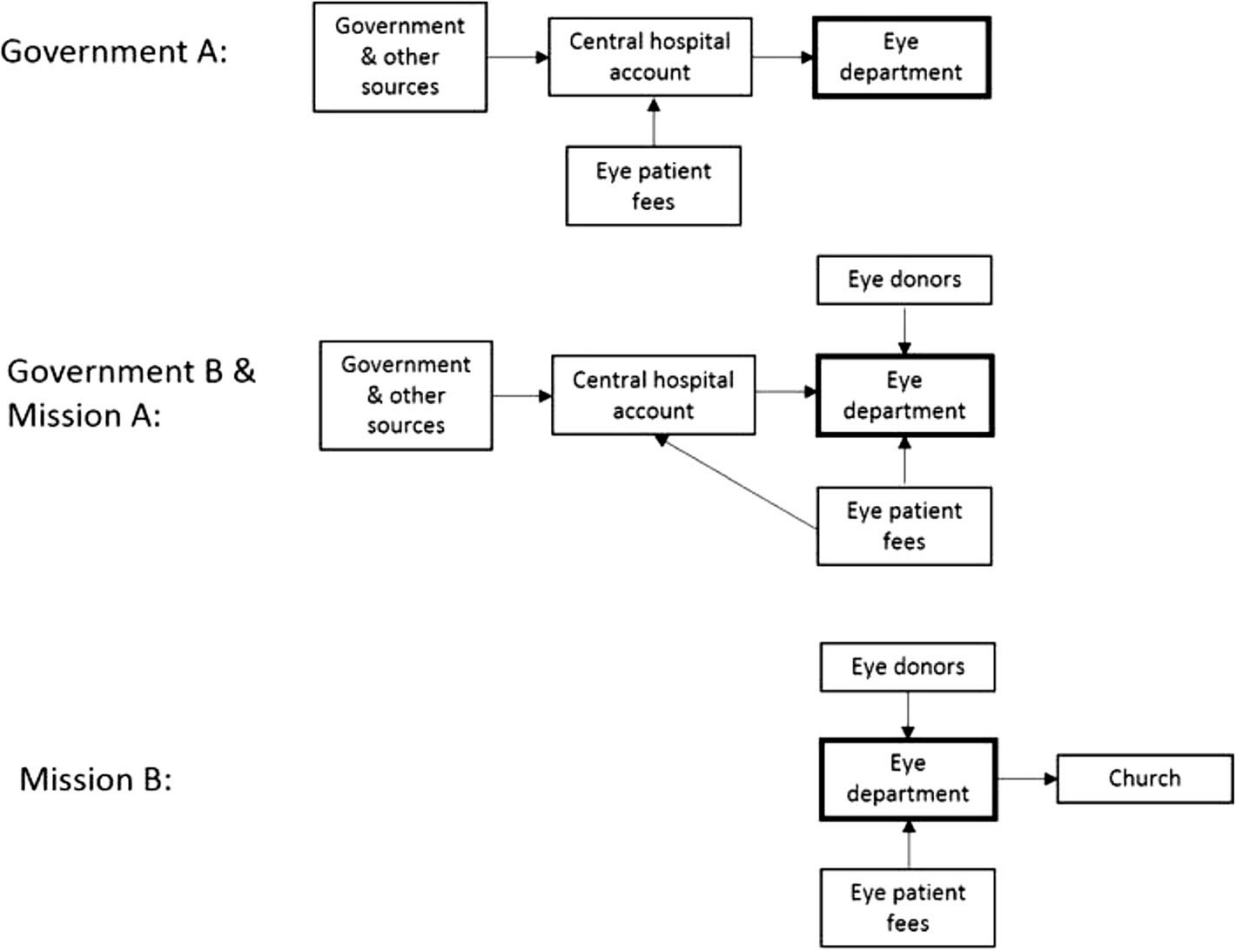
Schematic of revenue streams available to eye departments in case study hospitals in 2012

In 2012, three out of the four eye departments we studied had ‘sustainability funds’. Government Hospital A did not, and relied entirely on the hospital to provide income for consumables and any other purchases they needed (Fig. 1). As all other teams received donor funding, it is possible that the verticality of donor accounting processes helped initiation of separately-controlled eye accounts in these hospitals. In both Government Hospital B and Mission Hospital A, a portion of patient fees revenue was given to the hospital to enable central purchases, but the eye department eventually received the value back in-kind through some consumables and access to hospital infrastructure. In the mission sector, teams had more financial independence; Mission Hospital A staff, for example, rarely had to negotiate permission to use funds for outreach activities or professional development expenses such as attendance at zonal or international meetings.

Mission Hospital B eye department’s income was the most partitioned. While it was required to contribute some income to the Church, it had recently opened a separate eye service-only fee collection window from the hospital’s and stopped contributing to the hospital’s central budget. From the perspective of the eye care unit, this was a protective measure since the hospital was near bankruptcy and, with the precariousness of eye care donor funding, patient fees were increasingly their largest source of income. Managed independently from the rest of the hospital, this system ensured that all eye patients paid before receiving treatment and funds were available immediately to pay for consumables, salaries and other service delivery costs. Mission Hospital B was a rare example of a department within a hospital that had the financial capacity to pay its staff salaries every month.

Over the study period, the eye team in Government Hospital A came to the conclusion that they needed to develop a sustainability fund, like in other successful eye hospitals they had observed in Tanzania. A 2013 visit by the eye team to Mission Hospital B was particularly influential. In the Government Hospital A team’s words, although Mission hospital B received less donor support now, larger amounts from CBM historically had helped them become “*a well-established unit. They were getting a good support before, then after they matured, they started to move themselves* […] *now they are managing themselves from patient fees. It is working*”. They judged that, as a government facility, they could not set-up their own fee collection window for the department. However, after this visit they became more systematic about ensuring patients had paid their fees, before administering treatment. This allowed them to independently track the eye unit income, collecting information on a ‘virtual’ sustainability fund, which they planned to use in future negotiations with hospital managers to demonstrate the (monetary) value of their service and therefore advocate for more hospital funds being spent in the eye department.

The circumstance differed in each case study, but ultimately the creation of this parallel financing system (where rules of collection, pooling and purchasing were solely determined by the eye care team in response to their specific needs) was a resourceful way for local actors to put into place new systems where the official government-donor partnership system had failed.

### Sustainability strategy 2: avoid exemptions

At the same time as many eye departments were working towards increasing their income from patient fees, however, practitioners concomitantly feared an over-reliance on patient fee collection if this came to be perceived by the population to be in contradiction with existing government poverty-reduction policies.

Although free healthcare was introduced to government facilities at Independence, severe budget deficits eventually led Tanzania to reinstate user fees in 1993 for all but a few categories of patients [46]. Given difficulties verifying age, carrying out economic means tests and shifting political narratives about the contribution of older people to national development, over-60s were also granted universal exemptions for healthcare a decade later [47–49]. This policy is particularly relevant to eye health services, since vision declines rapidly with age and users are mainly older people: globally, 82% of blind and 65% of visually-impaired people are over the age of 50 [50]. With no clear mechanism in place to account for or reimburse these exemptions, however, eye care practitioners widely saw this policy as practically problematic, commonly posing the question: “*We don’t see any compensation from the government* […] *Who is going to pay for them?*” (ECP at Mission Hospital A). Rather, adoption of this policy was mainly seen as a populist issue, used to appeal to voters across the political spectrum in Tanzania using “*sweet words*” (ECP at Government Hospital B) but with few provisions for implementation: “*In theory the government says that they will pay for those people but when we go to the higher management, the district team says that they have no budget for that,* […] *so it is remaining an exemption forever* […] *it is merely political, nowhere can you stand and say, you government, you say from this policy. The government is leaving us in an uncertain position.*” (ECP at non-case study hospital).

For some, this reality meant that the policy was not implementable, and paradoxically, again contributed to the overall government neglect of eye health at the policy level. As one practitioner put it, “*ours* [eye disease] *is a condition which is not involved in free care*” (*ECP at Government Hospital A*). Others, however, justified skirting this policy using sectoral arguments. Whereas practitioners in the government sector, if discovered, were at risk of public shaming in the media, mission sector hospitals, even district-designated ones, could claim parastatal status to explain their fee structure. However, moral justifications could be found for government hospitals too, since systemic government deficiencies made it difficult to implement any policy affecting eye care, such as the minimum human resources and equipment needed to deliver eye services: “*They* [government] *speak well but don’t put into practice* […] *what they write in policies, for example ensuring government hospitals have drugs, equipment and even simple things. This is why you find that mission hospitals have more patients than government hospitals*” (ECP at non-case study hospital). In practice, however, when challenged by patients, practitioners tended to justify their behaviour by explaining the financial deficit in their hospital and, under these circumstances, claiming relative modesty in their own pricing structure: “*Whereas in other places you would have to pay 100,000 plus eye drops, our rate is 40,000. If you pay 40,000 you will not be requested to pay for anything else*” (ECP, Government Hospital B).

In our study, we found no evidence that exemptions were routinely made for elderly eye care patients in any hospital in the Lake Region, including those with government health insurance at mission hospitals, since it took the hospital so long to be reimbursed. An exception was the multi-region outreach held on World Sight Day, which received separate donor funding and targeted ‘difficult to reach’ patients. Additionally, Government Hospital B started offering many more exemptions in the final month of study at the request of the hospital’s medical officer, who had received a personal visit from the Minister of Health. Furthermore, there appeared to be little collective appetite to address this complex problem transparently at LARESA meetings, despite recognition of its special importance to the eye care sector: a tentative proposal at one LARESA meeting to brainstorm solutions so that practitioners would not have to “*go against the law*” was shelved for future discussion (observation of LARESA meeting).

Ineffective implementation of exemption policies elsewhere in Tanzania has been previously explained by confusion about eligibility criteria as well as fear of jeopardising district funds’ financial viability [49]. Patient fees were the main source of income in the Lake Region case study hospitals (Table 2), which a universal exemption policy for the elderly would have put at substantial risk, threatening eye departments’ organisational viability. In fact, despite the prominence in eye health sustainability narratives of the concept of NGO relationships determining service performance, income from patient fees exceeded that from donors in each study hospital (Table 2). Additionally, since eye donors accounted for government contributions (and therefore sector ‘location’) in estimations of their annual disbursements, when income from both sectors was combined, eye departments with any donor actually received approximately similar amounts (TZS 28–32 million). The major differences in income generated overall were instead associated with the amounts departments accrued through patient fees (especially fees for surgeries).

**Table 2:** Sources of income and patient fees charged across eye departments by end of 2012

	Government A^a^	Government B^b^	Mission A	Mission B^c^
Sources of income (TZS)				
Government^d^	Unknown	17,180,000	7,900,000	0
Eye health donors	0	15,200,000	20,000,000	30,900,000
Patient fees	0	18,480,000	21,700,000	118,800,000
Total	Unknown	50,860,000	49,600,000	149,700,000
Patient fees charged for surgery	50,000	40,000	130,000	150,000

^a^Financial contributions could not be estimated by the cataract surgeon who did not participate in financial planning for the eye department in 2012. Income generation from eye surgeries began in 2013, so the 2013 patient fee value has been reported; patients also paid around TZS 4000 out of pocket on surgical consumables they were asked to provide privately

^b^This was the only hospital in the case study which sold spectacles (at TZS 20,000 per pair); this income has not been included in the analysis, for comparative purposes

^c^Disbursement of donor funding in Mission Hospital B was severely delayed in 2012; in response, patient fees were increased that year from 80,000 to 150,000. In Mission Hospital A, fees were also increased that year from 85,000 to 130,000 in response to rising hospital costs

^d^An unknown, small proportion of income classified as ‘government’ comes from patient fees using general hospital services

TZS, Tanzanian Shilling

Income from patient fees, however, was very dependent on the sector eye teams operated in. There was large variation in the amounts patients were charged for cataract surgeries across the study hospitals (ranging from TZS 40,000 to TZS 150,000; Table 2). Most actors interviewed could not name the exact amount charged by other facilities but knew generally that fees tended to be higher in the mission sector than the government, where fees are capped by law. Unlike practices used in response to exemptions policies, government hospitals were hesitant to avoid implementation and inflate fees. While surgical patient fees in mission facilities were around twice as high as those in the government sector at the beginning of 2012, by the end of the year, they were three times as much, potentially highlighting the greater autonomy of eye units in the mission sector to respond to economic threats affecting the sustainability of their services.

Therefore, contrary to actors’ outward impressions of sustainability in the Lake Region eye health system, eye departments in the mission sector appeared not to be financially better off because of their privileged access to donor financing (although this was a contribution), they were better off mainly because they had direct control (collection and management) of a revenue stream: patient fees, which contributed to a ‘sustainability fund’.

### Sustainability strategy 3: expand and contract services

Practitioners saw outreach as a necessary means to improving population eye health outcomes either by bringing surgical services closer to patients in remote areas (surgical outreach) or by screening and referring patients to services at central locations (clinical outreach). Both types of outreach helped sensitise communities to eye diseases and eye service availability, which generated demand for services, thereby promoting sustainability.

In 2012, there were some sectoral differences in the range of services available in eye departments (Table 3). Examinations and surgeries were both routinely offered at base facilities and via outreach by mission hospitals. In the government hospitals, surgical outreach services in particular were more limited. By the end of the study period, however, the government hospitals had taken promising steps to expand the types of services they offered, whereas services had contracted in one mission hospital (Box 1). Partly, this related to the ways eye teams individually viewed outreach in the trade-off between today’s needs and future sustainability, namely as too expensive (Mission Hospital B), as a risk to equipment (Government Hospital B) or as an opportunity for co-funding (Government and Mission Hospitals A).

**Table 3:** Changes in availability of services in case study eye departments, 2012–2013

Services available	Government A		Government B		Mission A		Mission B	
	2012	2013	2012	2013	2012	2013	2012	2013
Examinations at facility	Yes	Yes	Yes	Yes	Yes	Yes	Yes	Yes
Surgery at facility	No	Yes	Yes	Yes	Yes	Yes	Yes	Yes
Examinations via outreach	No	Yes	Yes	Yes	Yes	Yes	Yes	Yes
Surgery via outreach	No	Yes	No	Yes	Yes	Yes	Yes	Yes

Yes, service was available; No, service was not available

The experiences of three hospitals highlighted the unpredictability of donor funds for service expansion. Donors had made particular commitments which could only sometimes be adjusted or re-committed to other hospitals within the local eye health system as circumstances changed. Local governments, likewise, could only sometimes be cajoled into co-funding outreach.

In contrast, individual eye departments uniformly demonstrated flexibility and personal resourcefulness in response to changes in funding structures, even in long-running programmes. For example, Mission Hospital A had developed strategies to extract commitments for outreach funding from several local authorities in their region and income from both donors and patients contributed to their ability to change their activities quickly when even small amounts of government funding became available. The willingness of the Government Hospital A team to use personal income to pay the transportation costs for donated equipment, even before they had initiated their virtual ‘sustainability fund’, demonstrated an understanding that personal initiatives, in particular, were necessary to run an eye unit in this kind of health system structure, no matter the sector.

### Box 1. Evolution of cataract surgery services in each case study hospital

Mission A: The only hospital in the Lake Region to have previously been staffed by two cataract surgeons, the 2012 eye team inherited a very busy outreach schedule which they largely maintained during the study period, despite a halving in surgical capacity. The weekly outreach schedule was diverse, including visits to a school for albino and blind students, a prison, a leprosy camp settlement, and most hospitals in their own and surrounding regions. More surgeries were performed outside than inside their base hospital, either by travelling with operating equipment to use in district hospitals’ theatres or by using additional equipment centrally stored at a regional hospital lacking surgical ECPs. Patient fees charged at these clinical and surgical outreach visits were very flexible: while donor support covered fuel, travel costs and a portion of the supplies, the team was sometimes able to offer services for free, such as at the prison, when the Regional Medical Office agreed to help finance the activities, or at a subsidised rate in particular hospitals if the District Medical Office showed willingness to contribute some support such as team per diems. Changes to the schedule were also sometimes made to respond to and incentivise such local government contributions. The main factor constraining expansion appeared to be the lack of staff able to run both outreach and services at the central facility. Mission B: In previous years, this eye team had performed clinical and surgical outreach on a large scale with donor support, but a reduction in this funding combined with severe delays in the 2012 disbursement threatened the viability of their programme. The team therefore felt compelled to reduce their outreach activities to concentrate on raising income through patient fees at the hospital. As one ECP stated, “*if the CBM portion reduces, we simply reduce our* [outreach] *activities*”, since cash outlays are always needed in advance of these activities for fuel and per diems, in addition to the consumables normally stocked for base-hospital surgeries. Only very limited clinical and surgical outreach activities were maintained that year to honour long-standing agreements with two mission facilities which had received foreign donor funding for this purpose. The team could not, however, perform surgical outreach when requested to replace surgeons unable to travel from another NGO, as the NGO would not transfer funds for consumables and disagreed with the team charging patient fees to recoup this expense. The team instead organised a clinical outreach and referred eligible patients to their base hospital for surgery. This was the only team that did not seek financial contributions for outreach expansion from the local government.

Government B: Since 2005, the eye team had led outreach activities with external funding within one of the only official district-level ‘VISION 2020’ programmes in the country. By 2007, the number of surgeries performed annually began to plateau, at around 600. Although substantial numbers of patients were examined, all outreach to district facilities was clinical. Surgical outreach in the region had only been performed several years earlier by a visiting surgeon. In 2012, the team were reluctant to perform surgery during their own outreaches for fear of further damaging their operating microscope which had not been serviced since the donation was originally made and was in need of repair. It was not until after a 2012 LARESA meeting, when members discussed the importance of doing outreach, that the team re-considered performing surgeries and requested supplemental funding to do so from their eye donor, who also agreed to fund the microscope repair. Government A: In 2012, the hospital received a recently graduated cataract surgeon. Staffed until then by a retired surgeon, no surgeries had been conducted there in the previous 4 years due to that surgeon’s own deteriorating vision. The new surgeon had received his training in robust teaching hospital outreach programmes and so was disappointed on arrival when he found the hospital’s operating equipment rusted and in disrepair. A frustrating 12-month period of conversations with the training school, the national eye care programme and other donors ensued before a solution was found. The training school identified a donor for the equipment who had unallocated equipment funding for government hospitals; the cataract surgeon himself would pay for the equipment transportation costs, to be reimbursed later by the hospital. Sufficient consumables for 100 patients were also provided to seed the eye department’s ‘sustainability fund’ which could then be used to order more. Although the retired surgeon advised him to prioritise setting up a good service at the hospital before starting to do outreach, the new surgeon saw outreach as an activity that could attract co-funding for donations of consumables, transportation costs, even potentially equipment. Over this period he contacted eight types of actor about outreach or equipment, including several District-level Eye Care Coordinators and medical offices. CBM was unwilling to reallocate funds dedicated to another mission facility in the region which could not use them because this was a government hospital but funds from a different government-donor agreement that could not proceed could be used. When another NGO could not operate in one area of the region, the District Medical Office asked the new cataract surgeon to step-in, with additional financial support from their office, thereby adding limited outreach activities to this team’s portfolio of eye care services in 2013.

### Sustainability strategy 4: access peer support to ‘shout louder’

Eye care human resources in the Lake Region in 2012 were insufficient, but they were even scarcer in the 1990s. Then, there were only two cataract surgeons to serve the region, both supported by CBM (presentation by LARESA). Although they covered large distances, no one seemed to ‘know’ about cataract surgery services – not communities, not authorities, not hospitals, nor most other health workers. Eventually, through discussions with non-surgical eye care staff they met during their extensive outreach work, these surgeons realised that everyone, regardless of the sector they worked in, felt isolated, that “*every individual was just working independently*” (*ibid*) so they decided to create a network to bring everyone together. They reasoned, “*if we can organise ourselves, we can shout louder*” about the deprivation eye health suffered in their region. The primary objective was then to speak with one unified voice in order to attract more resources for eye care. Further, if global and national strategy goals were ever to be achieved in their area, Lake Region actors such as themselves would first need “*to collect all the problems, analyse them and present them* […] *for help or attention*”.

Their primary targets at this time were the Ministry of Health and Social Welfare and CBM. These bodies, however, refused to work with such an organisation, whose informal structure had little precedent in the country. From CBM’s perspective, they were “*not government, not mission, not a professional organisation, not an NGO*”, so for accounting purposes it would be more efficient to work through existing agreements with members’ mission hospitals (interview with CBM). From the Ministry’s, LARESA was accused of trying to separate Lake Region planning from federal processes to compete for donor attention (interview with LARESA chairperson). Its members demoralised, LARESA was disbanded for 12 years until a convergence of factors prompted its re-establishment in 2008.

It now works with the national control programme and donors to organise annual zonal-wide surgical camps, sensitise regional governments to eye care needs, collect information on equipment and training needs, and advocate for the fair distribution of these resources across facilities (LARESA 5 year strategic plan 2012–2017). Through less formal means, LARESA also seeks to counter professional isolation by offering the experience of its members during meetings as a technical resource to other members who need help solving problems in their day-to-day work. Peer coaching to solve problems and model alternative practices (for example, inspiration to track income for a future sustainability fund in Government Hospital A or advice to ask for microscope repair in Government Hospital B, above) seemed to us to have a very practical influence on adapting sustainability strategies in case studies during the study period.

Stemming from the above philosophy and origins, an important feature of this social network is its awareness of the different ways eye care works through government, mission and NGO structures as well as the shared problems ECPs across sectors must grapple with because of the systemic deficiencies of this long-standing partnership model. This explains the oft-repeated idea by LARESA members: “*whether you are from public or mission sector, we should all come together to work for the poor people of this region. If you are an eye health provider, you are automatically a member*” (ECP at LARESA meeting).

## Discussion

### Neglect as opportunity: spaces for decision-making in a decentralised system

Eye care, like other specialist hospital services in Tanzania, developed unevenly in the 20th Century as a result of wider health system structural changes. Today, in the north-west Lake Region, the most complex eye care activities are planned and performed by cataract surgeons who are disproportionately employed in mission-owned facilities. By the time of our study, remarkably coherent narratives about eye health had developed among practitioners who described eye care as ‘all under the NGOs’ because of the systemic neglect that eye care suffered by all levels of government. While eye health may have been ‘political’ during Nyerere’s literacy campaigns after Independence, by 2012, eye care practitioners had become distrustful of government to engage meaningfully in eye health development. This feeling of neglect became more acute as practitioners discussed the precariousness of support from international donors and missions. Against this narrative backdrop, using innovative health systems research methodologies, we explored how eye teams worked towards sustainability in this context of neglect, within a decentralised, plural health system.

While the official government eye health system in Tanzania may be designed to be hierarchical (whereby rules are made at the highest levels and passed down for health workers to follow), other forms of governance typically emerge when hierarchical systems fail [51, 52]. In this case (as elsewhere [25]), Church missions have provided an important organised form of ‘co-governance’, but in this study we have also highlighted the role that ‘self-governance’ plays when individual eye care units and the LARESA network have the space to create their own rules [51].

With these overlapping modes of governance co-existing in the same health system, the differences we observed between our four case study eye departments could not be completely explained by their position in a particular mission or government sector. In fact, we observed that teams in different hospitals found similar strategies to manage the sustainability of their programme even when their management structures or governance systems were unique. As has been found elsewhere [53, 54] this suggested to us that, within this weak, decentralised government structure, the multiplicity of rules, which became available to govern service delivery, meant that eye care practitioners in the Lake Region had substantial decision space within which to operate. The challenge for eye care actors was to selectively draw from all governance systems so that they benefitted eye care programmes without jeopardising their activities. Four strategies, in particular, pointed to systemic trends operating in this decision space which should be accounted for when considering how best to advance eye health sustainability in the Lake Region.

### New rules to survive

Two sustainability strategies were related to eye programme financing, namely maintenance of ‘sustainability funds’ and avoidance of user fee exemptions. Sustainability funds, or bank accounts which contained income from donors and patient user fees, were kept by three of the four eye departments to maintain financial autonomy from their host hospitals. Mission-based departments tended to have greater power over spending decisions and, having observed a funding model which relied on such a fund in the mission sector, by the end of the study period the fourth eye team in the government sector had taken steps to begin their own independent fund. Contributions to this fund from patient income were seen as essential to guarantee financial flexibility because they were reliably and immediately available through delivery of standard services; in all models, they also contributed greater overall amounts than either donor or government sources. All teams therefore sought to maximise income from this source, by raising fees, by avoiding granting exemptions or both. Teams operating from the mission sector, especially, had greater freedom to avoid government policies that could have limited this strategy, but all teams felt justified in doing so.

With little apparent government, mission or public interest in the eye health service needs of elderly patients, eye care practitioners had neither the support to implement government policies sustainably, nor did they face opposition in decisions to skirt them. Eye care practitioners are not alone in selectively implementing exemptions in Tanzania because of weak government capacity to monitor health regulations [49]. In many low-income settings, in fact, informal rules or norms supersede formal mechanisms when these mechanisms are untenable, when there are no rules or when the rules are vague [55, 56]. They are a way of solving important problems such as a lack of basic drugs in hospitals [57] or how to sustain financing for eye care services. Indeed, some health systems research suggests that compliance with upstream bureaucratic accountability mechanisms can constrain local level innovation by front-line providers which may be needed to improve quality of care, responsiveness and accountability to patients [58]. Seen in this light, strategies to maintain sustainability funds and avoid exemptions appear to be supply side innovations that developed in the mission sector, were passed on to others in government hospitals and became routine, local norms followed by LARESA members which now act to morally ‘filter’ formal policies thereby influencing implementation behaviour [56].

Contradictory financial considerations also underlay the third sustainability strategy, namely to maintain willingness to expand and contract services. As financial circumstances worsened for one team, they elected to contract outreach services in order not to detract from higher-earning services at their base. This has probably been the most common experience of outreach health service provision funded by FBOs described in Tanzania [59]. In contrast, two other departments saw outreach as an opportunity to diversify their income. Both types of experience illustrated an inherent characteristic of outreach that limits the ability of actors to adopt it as a new activity or continue outreach after a ‘shock’ to the system such as withdrawal of donor funding: its low ‘compatibility’ with existing health systems [15]. Since outreach requires teams to work outside their typical place of work and interact with external actors, conducting outreach may not always be coherent with the perceived ‘mandates’ or expectations of other actors operating within the same governance systems so significant modifications to actor roles may be required to solve new problems such as access to transportation and equipment without donor support. In turn, accessing peer support and other forms of social capital through the fourth sustainability strategy was an important strategy to solve problems such as teams’ desires to conduct outreach.

In the long term, coherence (rather than standardisation) between the various governance systems in the Lake Region identified here will need to improve so that survival strategies of eye care providers do not endanger financial access of eye care services for populations.

### Eye care providers as social entrepreneurs

A major problem characteristic of pluralistic systems such as the Lake Region’s is that actors tend to work in isolation [60]. Currently, key resources such as equipment and donor contributions to outreach are partitioned by sector. This has contributed to the diversity of service delivery models we have already described, but it also limits the sustainability of the entire system. With little sharing of resources across the network, sustainability can only be described as the sum of the sustainability of each eye department within it, rather than a single, robust system which benefits from synergism.

This is why the formation of LARESA, the informal peer network which invites eye care practitioners, regardless of cadre and sector, to address eye health system sustainability through information gathering, experience sharing and advocacy, is important. As a multi-sector network, LARESA can help write new informal rules, reform official ones and establish beneficial norms by encouraging the ‘social entrepreneurs’ among its members (those who seek to create social value or social justice) to mentor others, for example [61–63]. That eye care providers in the Lake Region innovate mainly as individuals rather than as organisations should also be seen as beneficial. This contributes to their ability to innovate as they are detached from the constraints of traditional governance structures, allowing them the freedom to operate and reach groups that traditional governance models cannot [64, 65]. As Christian FBOs in Tanzania have tended to avoid advocacy which could be construed as political [66], the LARESA network could therefore be a more effective organisation to address controversial issues such as the over-60s exemption policy. A social entrepreneurship model has also been suggested by others in eye health to both spur and provide technical oversight of commercial initiatives to bring ready-made spectacles to rural areas in Africa and Asia, and which ultimately contribute to poverty alleviation [67].

## Conclusions

Can we say anything about the specific legacy of NGO, FBO and mission sector engagement with eye care in this part of Tanzania? What does Christian faith-based eye health work do for FBOs, churches and health systems? We used health systems research methodologies to track social dynamics crossing the public and non-profit private sectors to partially answer this question. Certainly, FBO structures are important. All teams relied on donors for access to equipment, and often this was through international mission structures, so mission-based eye care departments were at an advantage. These donors also supported surgical running and other costs in some facilities; this was critical in Mission Hospital B, the only eye department without access to any government resources for eye care. The importance of donor contributions was also highlighted in Government Hospital A, where services were extremely limited in the first 12 months of operation because of a lack of donor relationships. Ultimately, the most productive team with the most diversified service delivery (Mission Hospital A) drew financial and social capital from both the mission and government sectors.

Although some eye care donors in Tanzania may be particularly inspired by their faith, we found no evidence to suggest that eye health was particularly prioritised in local mission hospitals in the Lake Region. Indeed, rather than promoting local charity, either in-line with religious beliefs, Nyerere’s historical declaration of self-reliance or with the current government’s poverty-reduction provisions for older persons, the particular Church running Mission Hospital B itself actually received contributions from the eye department which ultimately came from patients.

Like other areas of health in Tanzania, eye health development has undoubtedly been held back by a legacy of deliberate under-funding by both government and Christian mission sectors in an attempt to prompt the other to fill that gap [59]. Can a mission health programme that extends access by charging triple the government-mandated patient fee really be considered pro-poor, as Christian FBOs typically purport to be [2]? However, as we have explained, this gap also created an informal space for innovation which benefits the current eye health system. By operating from missions, teams are allowed greater freedom to avoid implementing government policies which poorly address implementation realities in a system of decentralised eye services provision. Furthermore, these alternative ways of delivering services serve as models for government hospitals. An FBO like CBM’s investment is therefore positive not only in terms of the population health outcomes achieved by the surgical teams they have supported, but also in terms of the organisational models its partners have developed, which, through social networks such as LARESA, help to open practitioners’ eyes about what might be possible through observation of diversity. Just as missions originally invested in expatriate ophthalmologists in the 1970s to develop training programmes and eye care networks across the country, support to LARESA now offers an innovative opportunity to invest in social entrepreneurs dedicated to developing local eye care services and who, importantly, cut across eye health sectors in Tanzania.

Looking to the future, we sound a final note of caution: while separating eye department procedures from hospital systems and raising patient fees is an attractive solution to management problems due to neglect in the short term, in the long term, these strategies endanger the sustainability of the whole system. Ultimately, patients will be the ones to pay for a less efficient system and high user fees reduce population access and equity. Expanding insurance programmes and increasing government investment in mission-owned facilities through district-designated schemes may offer some resistance to these negative trends.

The effects of neglect in eye health appear to be more complex than we commonly realise, because, as the experience of practitioners in the Lake Region shows, neglect generates new dynamics that affect sustainability in unexpected ways.

## Additional file

Additional file 1: Indicators selected by LARESA members to monitor progress towards eye health system sustainability. (DOCX 13 kb)

## Abbreviations

AMOO: Assistant medical officer in ophthalmology
CBM: Christoffel-Blindenmission
ECP: Eye care practitioner
FBO: Faith-based organisations
LARESA: Lake Region Eye Care Services Association
NGO: Non-governmental organisation

## Footnotes

1 This ‘Lake Region’ name was defined for historical reasons by eye care practitioners from this area themselves who sought to describe their organisation of eye care practitioners as serving the four regions officially recognised as encompassed by the ‘Lake Zone’: Kagera, Mwanza, Musoma and Shinyanga, as well as three poorly-served neighbouring regions that Lake Zone eye care practitioners traditionally provided outreach to: Tabora and parts of two new regions, Simiyu and Geita.

2 The number of patient examinations was probably explained by several factors, including the size of the catchment population and patient demand for eye and/or general health services at the hospital which have multiple determinants not specifically explored in this study. For example, while it might have been expected that the team at Mission Hospital B would examine the fewest patients since the hospital served the smallest catchment population, Mission Hospital A’s catchment was smaller than the two government regional hospitals but they examined the most.
