# Supplementary material for: Circumventing ‘free care’ and ‘shouting louder’: using a health systems approach to study eye health system sustainability in government and mission facilities of north-west Tanzania
Source: Health Res Policy Syst. 2016 Sep 9;14(1):68. doi: 10.1186/s12961-016-0137-9 (PMC5017067; doi:10.1186/s12961-016-0137-9)
Supplement: Additional file 1: — Indicators selected by LARESA members to monitor progress towards eye health system sustainability. (DOCX 13 kb) [file 12961_2016_137_MOESM1_ESM.docx]

**Supplementary data: Indicators selected by LARESA members to monitor progress towards eye health system sustainability**

| **#** | **Sustainability indicator** | **Sustainability Target** |
| --- | --- | --- |
| **Component 1: Population health outcomes** | | |
| 1.1 | Number of cataract surgeries performed in the facility | 1,000 |
| 1.2 | Number of cataract surgeries performed at all facilities in the region | 1,000 |
| 1.3 | % of cataract surgeries in region performed via outreach | 60% |
| 1.4 | % of districts in region where at least 200 cataract surgeries were performed in a year (via outreach or static clinics) | 100% |
| 1.5 | Number of eye patients attended in the facility | 5,000 |
| 1.6 | % of districts in the region where at least 5000 eye patients were attended (considering data by teams from all facilities) | 100% |
| 1.7 | % of diabetic patients diagnosed at facility screened for diabetic eye condition | 90% |
| **Component 2: Quality & access of health services delivery** | | |
| 2.1 | % of patients who are satisfied with services that are provided in health facility | 75% |
| 2.2 | % of eyes operated for cataract with best corrected visual acuity of 6/18 or better after surgery | 80% |
| 2.3 | % of patients being consulted within 2 hrs of coming to the health facility | 75% |
| 2.4 | % of minimum number of eye care staff required by the National Eye Care Programme employed by the facility | 75% |
| 2.5 | % of appropriate infrastructure and functional equipment required by the National Eye Care Programme in the facility | 80% |
| **Component 3&4: Organisational capacity/viability of local authorities, local organisations & service providers** | | |
| 3.1 | Eye unit income from patient user fees | None chosen |
| 3.2 | Total eye unit income | None chosen |
| 3.3 | % of eye unit income from user fees | 60% |
| 3.4 | Number of quarters when money has been timely disbursed to eye care unit | 4 |
| 3.5 | % of funds disbursed compared to requested budget | 100% |
| 3.6 | Budget line for eye care activities exists at the facility level | Yes |
| 3.7 | % of eye care providers who work full time in eye care unit | 100% |
| 3.8 | Number of quarterly meetings held with eye unit staff per year | 4 |
| 3.9 | Number of annual facility planning meetings where eye care staff is involved | 1 |
| **Component 5: Community capacity** | | |
| 5.1 | Number of sensitization meetings conducted by facility per year | 4 |
| 5.2 | Number of community feedback meetings conducted by facility per year | 4 |
| 5.3 | % of villages in the region which have a trained community eye worker | 100% |
| **Component 6: Enabling environment** | | |
| 6.1 | % of population covered by health insurance | 50% |
| 6.2 | % of government budget allocated to health | 15% |
| 6.3 | Number of strikes by government health staff | ≤3 |
| 6.4 | % increase in the national Human Development Index | Any increase |
